# Supplementary material for: One Brain—All Cells: A Comprehensive Protocol to Isolate All Principal CNS-Resident Cell Types from Brain and Spinal Cord of Adult Healthy and EAE Mice
Source: Cells. 2021 Mar 15;10(3):651. doi: 10.3390/cells10030651 (PMC7999839; doi:10.3390/cells10030651)
Supplement: Supplementary file 1 [file cells-10-00651-s001.zip › cells-1067670_revised Supplementary Figures_revision2/cells-1067670_Revision_Supplementary Figure Legends_V4.docx]

**Supplementary Material:**

**Figure S1. Further validation of the applied gating strategies *via* FMOs, immunocytochemistry of the isolated microglia fraction (CD45^int^CD11b^high^ cells) and Ly6C/G staining. (a)** Overlap of FMOs and flow cytometry stainings of the four isolated CNS-resident cells with their respective cell-type specific markers. **(b-e)** Immunocytochemistry of microglial markers Iba-1 (b) and CD11b (d). Negative controls (c, e) confirmed specificity of both primary antibodies. Fluorescence images were acquired with a Zeiss Axio Scope.A1 using 40- (d, e) and 63-fold (b, c) objectives and optimal exposure times. **(f, g)** Flow cytometry analysis of Ly6C (f) and Ly6G (g) expression levels by isolated microglia. Neutrophils (gated as CD45^high^CD11b^+^) were used as positive controls, while the respective FMOs served as negative controls. Low Ly6 expression levels by the isolated microglia confirmed their purity negating contamination by other CNS-resident myeloid populations (e.g. monocytes, macrophages, dendritic cells, natural killer cells and granulocytes).

**Figure S2. Flow cytometry analyses of oligodendrocytes and astrocytes isolated from adult EAE mice. (a)** Purity analysis of oligodendrocytes isolated from EAE mice *via* anti-O4 Microbeads by reference to a representative replicate. 92.37 % of the isolated cells expressed the oligodendrocyte specific surface marker O4. **(b)** Exemplary purity analysis of astrocytes derived from adult EAE mice after depletion of oligodendrocytes showing a viability of 77.34 % and a purity of 77.44 %.

**Figure S3. Flow cytometry analyses of neurons isolated from adult EAE mice.** Purity analysis of neurons isolated from EAE mice depleting all non-neuronal cells by biotinylation and consecutive magnetic labeling. In the depicted replicate, 75.55% of all isolated cells were viable, 89.53 % of these cells expressed the neuronal specific nuclear protein NeuN.
