# Supplementary figures and images for: One Brain—All Cells: A Comprehensive Protocol to Isolate All Principal CNS-Resident Cell Types from Brain and Spinal Cord of Adult Healthy and EAE Mice
Source: Cells. 2021 Mar 15;10(3):651. doi: 10.3390/cells10030651 (PMC7999839; doi:10.3390/cells10030651)

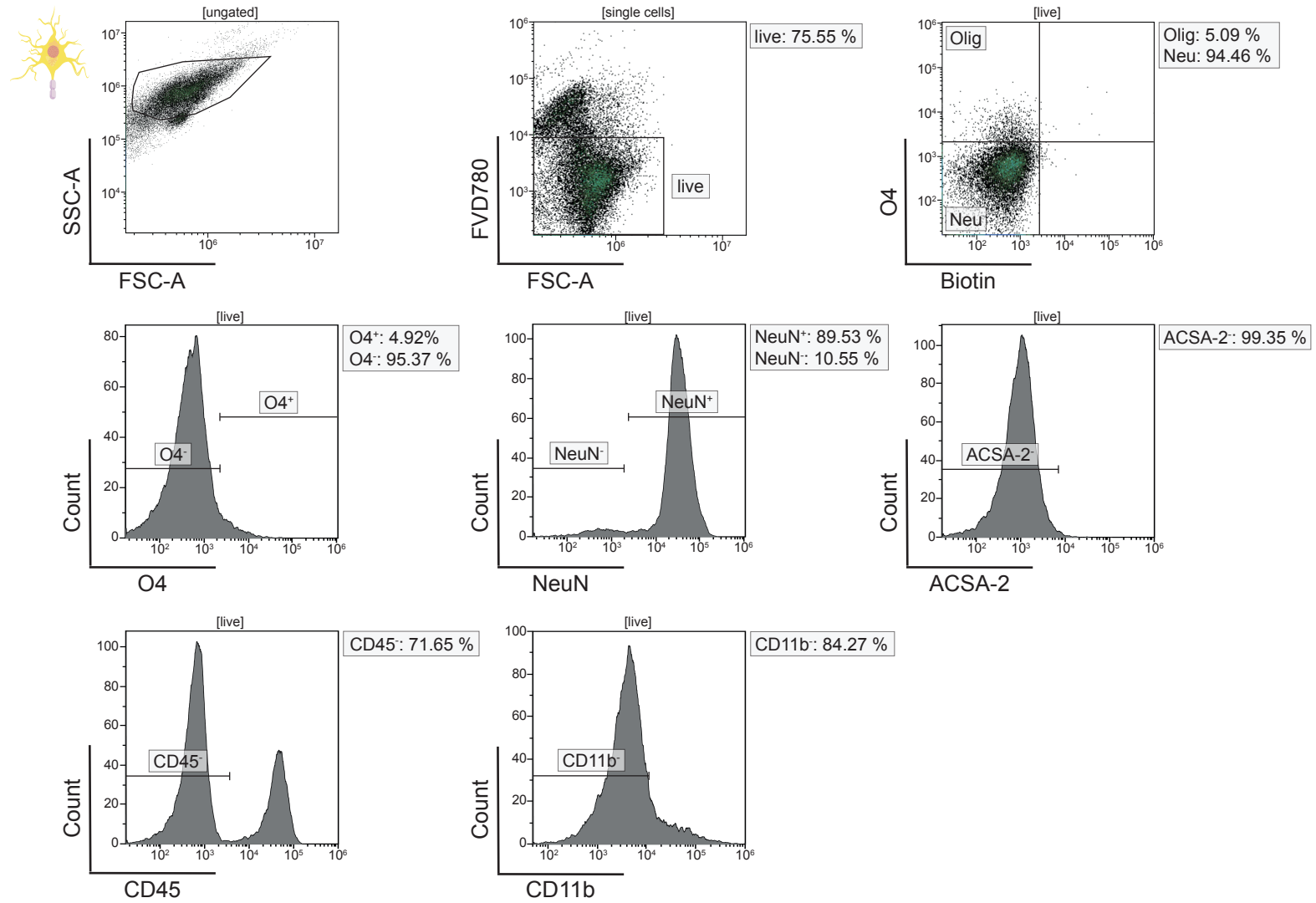

Figure S3

Supplement: Supplementary file 1 [file cells-10-00651-s001.zip › cells-1067670_revised Supplementary Figures_revision2/Methods Paper_Supplemental Figure 3.pdf]

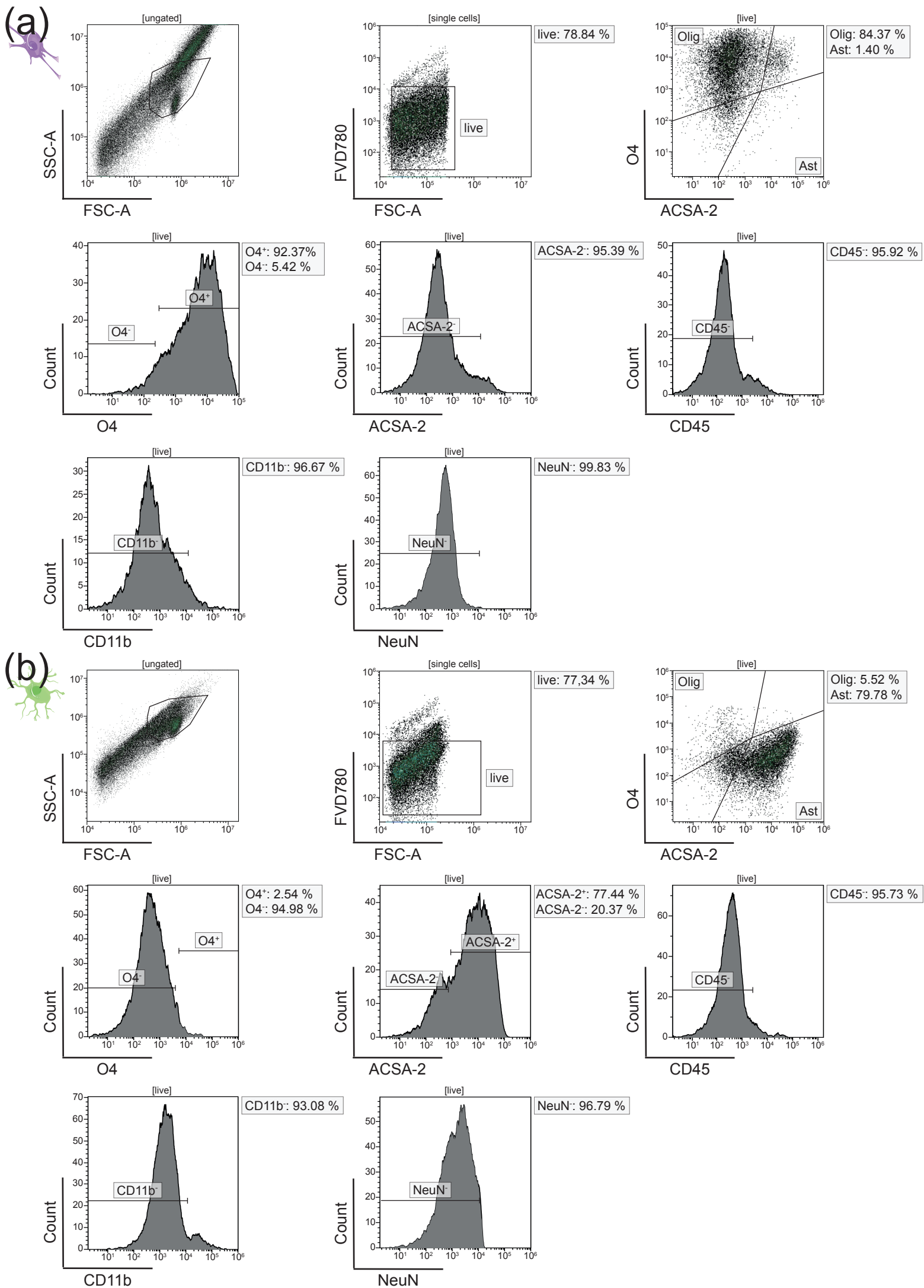

Figure S2

Supplement: Supplementary file 1 [file cells-10-00651-s001.zip › cells-1067670_revised Supplementary Figures_revision2/Methods Paper_Supplemental Figure 2.pdf]

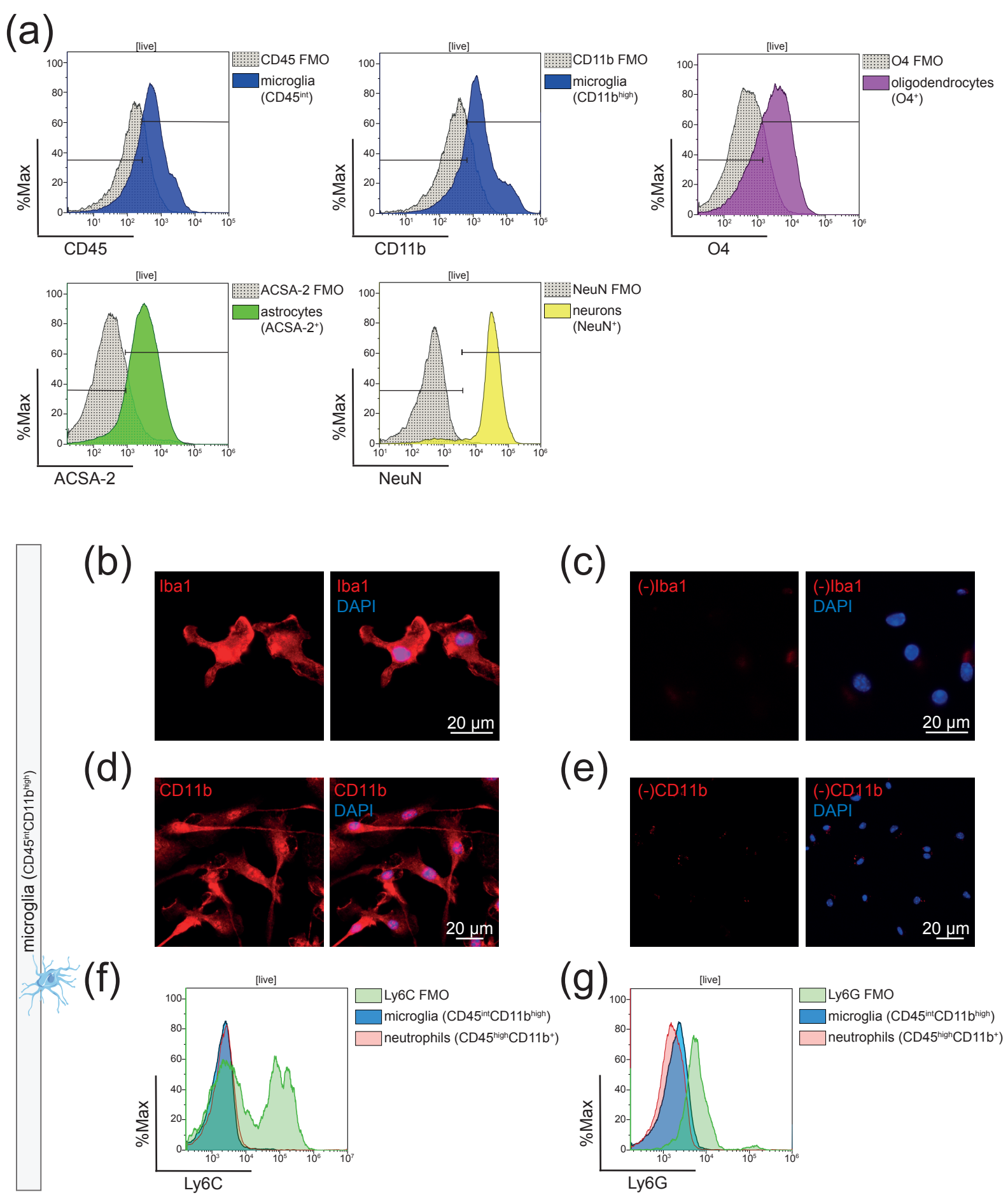

Figure S1

Supplement: Supplementary file 1 [file cells-10-00651-s001.zip › cells-1067670_revised Supplementary Figures_revision2/Methods Paper_Supplemental Figure 1_revision2.pdf]
